# A Mobile-Based Mindfulness and Social Support Program for Adolescents and Young Adults With Sarcoma: Development and Pilot Testing

**DOI:** 10.2196/10921

**Published:** 2019-03-18

**Authors:** Elizabeth Donovan, Sarah R Martin, Laura C Seidman, Lonnie K Zeltzer, Tara M Cousineau, Laura A Payne, Meredith Trant, Marjorie Weiman, Marla Knoll, Noah C Federman

**Affiliations:** 1 Department of Psychology College of Natural, Behavioral, and Health Sciences Simmons University Boston, MA United States; 2 BodiMojo, Inc Boston, MA United States; 3 Pediatric Pain and Palliative Care Program, Department of Pediatrics, David Geffen School of Medicine University of California Los Angeles, CA United States; 4 Department of Pediatrics, David Geffen School of Medicine University of California Los Angeles, CA United States; 5 Department of Care Coordination, Mattel Children’s Hospital University of California Los Angeles, CA United States

**Keywords:** cancer, mindfulness, social support, mobile app, adolescents, young adults

## Abstract

**Background:**

Approximately 70,000 adolescents and young adults (AYA) are diagnosed with cancer each year in the United States. Sarcomas carry a particularly high symptom burden and are some of the most common cancers among AYA. Recent work has documented significant levels of unmet needs among AYA with cancer, particularly the need for psychosocial support. Mobile technology may be a cost-effective and efficient way to deliver a psychosocial intervention to AYA with cancer and cancer survivors.

**Objective:**

The two aims of this study were to (1) develop a pilot version of a mobile-based mindfulness and social support program and (2) evaluate program usage and acceptability. An exploratory aim was to examine change in psychosocial outcomes.

**Methods:**

Thirty-seven AYA with sarcoma or sarcoma survivors, parents, and health care providers participated in the study. Semistructured interviews were conducted with 10 AYA, parents of five of the adolescents, and six health care providers. Themes from the interviews helped to inform the development of a mobile-based mindfulness pilot program and a companion Facebook-based social support group. Twenty AYA consented to participate in a single-arm pre-post evaluation of the program; 17 downloaded the app and joined the Facebook group. Seven of these participants had participated in the semistructured interviews. Six additional health care providers consented to participate in the evaluation stage.

**Results:**

On average, participants completed 16.9 of the 28 unique sessions and used the mindfulness app for a mean 10.2 (SD 8.2) days during the 28-day evaluation period. The majority of participants (16/17) engaged in the social group and posted at least one reply to the moderator’s prompts. The mean number of responses per person to the moderator of the social group was 15.2 of 31 (49%, range 0%-97%). Both AYA and health care providers responded positively to the Mindfulness for Resilience in Illness program and offered useful recommendations for improvements. Exploratory psychosocial analyses indicated there were no significant differences from pretest to posttest on measures of perceived social support, mindfulness, body image, or psychological functioning.

**Conclusions:**

This study offers preliminary support for the feasibility and acceptability of a mobile-based mindfulness and Facebook-based social support program for AYA with sarcoma. The feedback from AYA and health care providers will assist in creating a fully developed intervention.

**Trial Registration:**

ClinicalTrials.gov NCT03130751; https://clinicaltrials.gov/ct2/show/NCT03130751

## Introduction

The past decade has seen an emerging interest in identifying and meeting the unique needs of adolescents and young adults (AYA) with cancer [1,2]. Approximately 70,000 AYA are diagnosed with cancer each year in the United States, with the incidence of cancer in this cohort increasing steadily over the past 30 years [3]. Sarcomas are some of the most common cancers in AYA and are particularly difficult to cope with given the high symptom burden (eg, amputation, limb-salvage surgery) [4].

Those AYA diagnosed with cancer have a number of unmet psychosocial needs [2,5,6]. Living with uncertainty about the future can be an ongoing source of stress and anxiety during and after cancer treatment [7-9]. In addition, problems with maintaining or making new social relationships have often been cited as pressing issues for AYA with cancer [10-12]. Given the psychosocial challenges evident in this patient population, the Institute of Medicine National Cancer Policy Forum Workshop emphasized the need to address the unique developmental needs and quality of life in AYA with cancer [2]. Unfortunately, very few studies of psychosocial interventions for AYA with cancer have been conducted [13].

Two approaches that show promise for helping AYA to cope with the daily psychosocial challenges of cancer treatment are mindfulness and peer social support. Mindfulness-based interventions (MBIs) teach individuals how to observe thoughts and emotions in a nonjudgmental and compassionate manner and redirect attention to the present moment [14]. Mindfulness skills training may help AYA cope with thoughts and feelings rooted in the uncertainty of their prognosis [15] and distress surrounding treatment, adverse effects, and body image concerns. Indeed, among AYA who have finished cancer treatment, those who report higher mindfulness report significantly less distress and uncertainty than those who report lower mindfulness [16]. To date, very few studies have focused on the effect of MBIs in AYA with cancer. However, AYA cancer survivors who completed an 8-week MBI showed a significant reduction in emotional distress and improvement in quality of life, a significant reduction in negative attitudes toward self, and a significant improvement in mindfulness skills at a 3-month follow-up [17].

Given the lack of peer social support experienced by AYA with cancer, interventions that seek to connect patients or survivors with other AYA who have experienced cancer also show promise [18] because they give AYA the opportunity to receive support from someone who can understand their unique life-changing experience. Despite the documented need for social support in AYA with cancer, research on social support interventions are lacking in this population [18,19]. Olsen and Harger [20] examined the feasibility of implementing a nurse-led supportive social networking group for AYA with cancer; participants reported experiencing an increased sense of support following the intervention.

Young adult cancer survivors have expressed the need for interventions that are delivered remotely and provide social support [21]. MBIs are increasingly being delivered through mobile phones. A meta-analysis designed to estimate the overall effects of online MBIs (including mobile app-based MBIs) on mental health for a range of populations found that they had a moderate effect on stress and a small but significant beneficial impact on depression, anxiety, well-being, and mindfulness [22]. In addition, social media channels such as Facebook have been explored as a way to connect patients managing health conditions with one another. Key recommendations include iterative content development with input from the target patient population, exploring the role of group “champions,” complying with social media policies of health care institutions, and using comprehensive evaluation strategies [23].

This study had two aims: (1) develop a pilot version of the Mindfulness for Resilience in Illness intervention, a mobile-based mindfulness program and a companion Facebook-based social support program for AYA with sarcoma and survivors, informed by interviews with AYA who have experienced sarcoma, parents, and health care providers; and (2) conduct a single-arm 28-day pilot study to examine program usage and acceptability. An exploratory aim was to examine the effect of the Mindfulness for Resilience in Illness intervention on psychosocial outcomes (ie, mindfulness, social support, psychological functioning, and body image).

## Methods

### Aim 1: Development of the Pilot Program

The objective of this aim was to conduct semistructured interviews to inform the development of the Mindfulness for Resilience in Illness program.

#### Design

Our overall qualitative approach was deductive, informed by a preexisting Resilience in Illness Model that described factors associated with resilience in illness [24,25] in AYA with cancer.

#### Participants

Those AYA aged 13 to 25 years, previously diagnosed with sarcoma and either undergoing active treatment or within 5 years of transitioning to survivorship care, were eligible to participate. To capture a range of perspectives, we purposefully sought a representative sample of AYA in terms of age, gender, and treatment history (eg, on and off treatment, limb-salvage procedure, amputation). Inclusion criteria for parents were having an adolescent (aged 13-17 years) son or daughter enrolled in the study and being fluent in English. Given that there was likely to be variability in the living situation of young adults (eg, living independently or at college), we limited our parent interviews to parents of adolescents aged 13 to 17 years. For these interviews, we sought parents’ perspectives surrounding their adolescent’s day-to-day challenges. Health care providers currently providing clinical services to AYA with sarcoma were eligible to participate. Health care providers were purposefully recruited to represent the range of roles in a treatment team (eg, oncologists, nurses, and social workers).

#### Recruitment

The AYA and adolescents’ parents were recruited from a pediatric sarcoma clinic in Los Angeles, California. Research team personnel distributed research flyers to families waiting for clinic appointments and screened interested patients or parents over the phone. Eligible AYA and parent participants provided online assent and consent prior to beginning study procedures. Potential health care provider participants were identified from author NF’s network of providers. Author NF emailed study invitations to providers, and interested providers were contacted by research personnel. Providers gave oral consent over the phone prior to beginning study procedures.

#### Development of Interview Guides

We developed semistructured interview guides to explore ways in which a mobile-based mindfulness and social support program can address the psychosocial needs of AYAs with cancer during and posttreatment. Three separate interview guides were created for AYA, parents, and health care providers. The first section of the interview guides was informed by the Resilience in Illness Model (RIM) [24,25], which identified psychosocial factors associated with resilience in AYA with cancer (ie, spiritual perspective, social integration, family environment, coping skills, and hope-derived meaning). This model was chosen to help identify potential resilience-enhancing program components. The second section of the interview guides focused on the use of mobile technology and preferences surrounding potential program content (ie, mindfulness and Facebook social support component). First drafts of the semistructured interview guides were created by author ED and were revised with significant input from the research team. To facilitate the comparison of multiple perspectives on the same topics, the parent and provider interview guides mirrored the patient interview guide and included queries about their beliefs about AYAs’ needs for a mobile-based psychosocial intervention. Interview guides are available on request.

#### Procedure

Two female research team members with qualitative research experience conducted the interviews. No interviewer and interviewee had a prior relationship. Patient and parent interviews were conducted in-person (at the research study’s hospital-based offices) or by phone. Health care provider interviews were conducted by phone. All interviews lasted between 45 and 90 minutes. Interviewees were offered Amazon gift cards of US$25 (AYA and parents) or US$100 (providers) as compensation for participating in the study. The research team continued to collect interview data until 10 AYA, five parents of adolescents, and six providers had been interviewed. This coincided with when the team reached a consensus regarding thematic saturation. All interviews were audio recorded, transcribed verbatim, and deidentified for analysis. Interviews were conducted from December 2016 to March 2017. The study was approved by the University of California, Los Angeles Institutional Review Board (IRB).

#### Data Analysis

The coding scheme was created by the research team. We used deductive coding, informed by the RIM model [24,25]. As such, author ED created an initial coding structure based on the interview guide, with each factor in the RIM model represented. In addition, codes were created to capture beliefs about the proposed program delivery method (mobile device) and program content (mindfulness and social support). Three research team members trained in qualitative analysis coded the interview transcripts in duplicate and discrepancies were resolved with discussion. Coded interview transcripts were entered into NVivo software (version 10) [26]. Two members of the team independently reviewed data associated with each code and met weekly to discuss emerging themes, ending the discussion when three to five themes were agreed upon. Themes, supporting quotes, and exceptions to the themes were entered into an Excel sheet.

### Aim 2: Pilot Test of the Mindfulness for Resilience in Illness Program

The aim of the pilot study was to evaluate the Mindfulness for Resilience in Illness mobile-based mindfulness and Facebook social support program for AYA with sarcoma by assessing usage and acceptance and exploring psychosocial outcomes.

#### Design

The study consisted of a single-arm, pre-post evaluation of the pilot program.

#### Participants

The inclusion criteria for AYAs and providers used for the formative interview phase were also used for the pilot test. Parents were not included in the pilot phase of the study. Participants included 17 AYA, seven of whom had participated in the qualitative interviews, and six health care providers.

#### Recruitment

The AYA were recruited from the same sarcoma clinic from aim 1. The same recruitment method used during the formative interview stage was used to recruit AYA and provider participants for the pilot study.

#### Mindfulness for Resilience in Illness Intervention Program

The 4-week Mindfulness for Resilience in Illness program consisted of a mindfulness mobile app, a private Facebook group, and a provider guide. The mindfulness mobile app was delivered although a customized version of an existing mindfulness mobile app (Whil Concepts, Inc) available through the Apple iOS App Store and Android Google Play Store. A total of 28 mindfulness audio exercises were selected from the Whil library for the 4-week program. The Learning to BREATHE framework [27] and themes from the qualitative interviews informed the selection of mindfulness meditations for the four weekly program themes: (1) breathe and listen to your body, (2) dealing with difficult emotions, (3) dealing with negative thoughts, and (4) being kind to yourself through challenging times (Textbox 1). Program host videos featuring two sarcoma survivors were also included in the app. The videos were used to familiarize AYA with mindfulness terms and introduce and conclude the weekly program exercises. Four weekly blog posts designed to reinforce the weekly mindfulness concepts were also created for the app and posted on the Facebook group.

The goals of the private Facebook group were to (1) solicit feedback about the app and (2) promote social support among group members. A sarcoma survivor was recruited to moderate the Facebook group, which included posting daily content and facilitating conversation among participants by responding to posts. A total of 38 messages were created for the moderator to post daily (one or two per day).

The provider guide (e-book) was developed to help providers support patients using the Mindfulness for Resilience in Illness program. The 12-page guide provided information about the rationale for teaching mindfulness skills to AYA with cancer and encouraging peer-to-peer social support, with specific strategies for supporting patients through each of the 4 weeks of the program.

#### Procedure

Both AYA participants and parents (if the AYA was younger than 18 years) completed online assent or consent forms via a secure online survey platform. Following assent or consent, participants also completed demographic questions and psychosocial measures (described subsequently) via the online survey platform. Before beginning the program, AYA participants were invited to attend a 20-minute webinar that provided (1) an overview of how to use the mobile app and the Facebook group and (2) an outline of the 4-week program components (eg, meditations, Facebook posts) and expectations (ie, one meditation per day for 28 days and responding to daily Facebook posts). All AYA participants began the 4-week study on the Monday after the week that the webinars were held. Psychosocial measures and an acceptance test were completed immediately following the completion of the program. The AYA were compensated with a US$75 Amazon gift card.

Three people were involved in the daily administration of the Facebook group: the moderator and two monitors. The moderator was paid US$25 per day for a total of US$750. In addition, two members of the hospital-based research team (authors SRM and LCS) served as monitors for the Facebook group. They were trained by authors LKZ and LAP, a pediatrician and clinical psychologist, to review posts on the Facebook group and to follow the emergency protocol in the case of code of conduct violations (eg, bullying) or signs of self-harm. No such incidents occurred during the study period.

After indicating interest in participating, health care providers were emailed the IRB-approved information sheet serving as informed consent. Providers were emailed the Mindfulness for Resilience in Illness provider guide e-book, instructions for downloading the app, and a link to the online acceptance test. Providers were compensated with a US$100 Amazon gift card. This pilot study was approved by the University of California, Los Angeles IRB and registered on ClinicalTrials.gov (NCT03130751).

**Description of the weekly sessions of the mindfulness program.**

**Week 1: Breathe and Listen to Your Body**

- Focus Your Attention
- Belly Breathing
- Finding an Anchor
- Fuller Deeper Breath
- 1, 2, 3 Breath
- Sense the Body
- Happy Breath

**Week 2: Dealing With Difficult Emotions**

- Welcome Emotions
- Feel to Heal
- Recognize Challenge
- Present With Anger
- Name It and Tame It
- This Too Shall Pass
- Discover A Safe Place

**Week 3: Dealing With Negative Thoughts**

- Recognize and Release Thoughts
- Unfuse our Beliefs
- Attention to Change
- Reflect and Redirect
- Dealing with Parents
- Paradox of Letting Go
- Skillful distraction (Exercise)

**Week 4: Being Kind to Yourself Through Challenging Times**

- Understanding Compassion
- Inward Compassion
- I Heart My Body
- Wishing Well
- Like Floating
- Simple Gratitude (Exercise)
- Circle of Kindness

#### Measures

##### Demographics

The AYA participants were asked about demographic characteristics during study enrollment, including age in years, race and ethnicity, and treatment status. Providers were asked their clinical specialty.

##### Usage

Use of the mindfulness app was assessed with (1) number of unique sessions completed (out of 28), (2) number of days using the mindfulness app, and (3) total minutes of engagement with the mindfulness app. Use of the Facebook group was assessed with the response rate to the daily posts.

##### Acceptance

The acceptance test included questions about general impressions of the mindfulness app, as well as the app’s features. Participants were also asked about the helpfulness of the app features and the Facebook group, using both Likert scales and optional open-text fields.

Providers were asked about the usefulness of the program for AYA, the usefulness of the provider guide, and whether they would recommend it to another health provider.

##### Psychosocial Measures

Mindfulness was measured with the 10-item Child and Adolescent Mindfulness Measure (CAMM) [28]. Items are rated on a 0 (never true) to 4 (always true) scale. The CAMM is scored by reverse scoring all items and calculating a sum. Total scores can range from 0 to 40 with higher scores indicating higher levels of mindfulness. The CAMM has demonstrated validity and reliability [28].

Social support was measured with the 20-item Perceived Social Support, Friends (PSS-Fr) scale, which assesses social functioning and peer support [29]. Answer choices for the PSS-FR are “yes,” “no,” and “don’t know.” Responses indicating positive perceived social support are scored as 1 (other responses scored as 0). Total scores range from 0 to 20 with higher scores indicating more perceived social support. The PSS-Fr scale has demonstrated validity and reliability [29] and has been used in AYA with cancer [30].

Psychological functioning was measured with the six-item Pediatric Cancer Quality of Life Inventory 32-Psychological Functioning subscale (PCQL-32-PF) assessing levels of fear, sadness, and worry, including those related to cancer symptoms and relapse [31,32]. Items are scored on a 0 (never a problem) to 3 (always a problem) scale, and answer choices are summed to create the subscale score. The PF subscale scores range from 0 to 18 with higher scores indicating higher impairment (ie, lower psychological functioning).

Body image was measured using three items from the Body Image Scale (BIS) [33]. This subscale was created from items 1, 8, and 9 from the original measure, which was developed for use with cancer patients. These three items were selected by the clinical team as a brief but representative measure of body image issues faced by AYA sarcoma patients. The items were “Have you been feeling self-conscious about your appearance?” “Have you been feeling the treatment has left your body less whole?” and “Have you felt dissatisfied with your body?” Items are scored on a 0 (not at all) to 3 (very much) scale, and answer choices are summed to create the subscale score. Scores for this subscale range from 0 to 9 with higher scores indicating higher levels of body image distress.

#### Data Analysis

##### Usage

Descriptive analyses (means and standard deviations) characterized session completion, daily app usage, app engagement time, and Facebook responses.

##### Acceptance

Means and standard deviations or proportions were calculated for the quantitative acceptance test responses. Qualitative acceptance test responses were reviewed several times by members of the research team, and main themes were discussed and recorded.

##### Psychosocial Measures

Change in psychosocial outcomes from pretest to posttest were analyzed using paired sample *t* tests.

## Results

### Aim 1: Development of the Pilot Program

#### Characteristics of Participants

Five adolescents and their parents and six young adults (>18 years) were approached and screened. Of the five adolescents and their parents screened, all were eligible and enrolled. Of the six young adults screened, six were eligible and five enrolled. One patient indicated interest but was lost to follow-up. Seven providers were approached and six were screened. One provider declined indicating they were too busy. In total, 21 participants were interviewed. The AYA (n=10) ranged in age from 14 to 23 years (mean 19.3, SD 3.4 years; 50%, 5/10 female). All AYA reported a current or past diagnosis of sarcoma, except for one patient who had another cancer diagnosis but was receiving treatment through the sarcoma clinic. Four of the five parent participants and three of the six providers were female. Participant demographic data are presented in Table 1.

#### Main Themes

Our exploration of the ways in which a mobile-based mindfulness and social support program can address the psychosocial needs of AYAs with sarcoma yielded three main themes: anxiety about reoccurrence, openness to mindfulness, and desire to connect with other patients.

##### Main Theme 1: Anxiety About Reoccurrence

Most parents, providers, and patients described that patients are often consumed with worry about the future. Patients described how they feared medical scans or became overly focused on symptoms because of underlying fears about an uncertain future. For example, one participant described:

Well, there’s always that impending doom feeling, you know, it’s like a hypochondriac, you become a hypochondriac because you know the implications. For someone having a stabbing pain in their abdomen might be like, “Oh, I probably, I wasn’t breathing correctly while I was doing this or I was, I don’t know, I ate something bad.” For a former cancer patient, it’s like, “Oh, my God. Maybe my kidney suddenly decided this is the time to go,” you know?

patient 07

**Table 1:** Characteristics of the adolescents and young adults (AYA), parents, and providers participating in the qualitative interviews (aim 1).

Characteristic		AYA (n=10)	Parents (n=5)	Providers (n=6)	Total (N=21)
Age (years), mean (SD)		19.3 (3.4)			
**Age range (years), n (%)**					
	13-17	5 (50)			
	18-25	5 (50)			
**Sex, n (%)**					
	Male	5 (50)	1 (20)	3 (50)	9 (43)
	Female	5 (50)	4 (80)	3 (50)	12 (57)
**Ethnicity, n (%)**					
	Hispanic/Latino	4 (40)	1 (20)	1 (17)	6 (29)
	Non-Hispanic/Non-Latino	5 (50)	4 (80)	5 (83)	14 (67)
	Unknown	1 (10)	0 (0)	0 (0)	1 (5)
**Race, n (%)**					
	White	6 (60)	3 (60)	5 (83)	14 (67)
	Black/African American	1 (10)	1(20)	0 (0)	2 (10)
	Asian	0 (0)	0 (0)	1 (17)	1 (5)
	Multiracial	2 (20)	1 (20)	0 (0)	3(14)
	Unknown	1 (10)	0 (0)	0 (0)	1 (5)
**Treatment status, n (%)**					
	On treatment	2 (20)			
	1-3 months posttreatment	2 (20)			
	4-8 months posttreatment	1 (10)			
	1-2 years posttreatment	3 (30)			
	3-6 years posttreatment	2 (20)			
	Off treatment for unknown duration	0 (0)			
**Providers’ specialties, n (%)**					
	Oncologist			3 (50)	
	Nurse or medical assistant			2 (33)	
	Child life, psychology			1 (17)	

##### Main Theme 2: Openness to Mindfulness

When asked about interest in mindfulness to cope with anxiety, most AYA expressed being open to mindfulness but were generally unfamiliar with mindfulness skill-building strategies. A young participant described her interest in learning more about mindfulness skills (eg, present moment awareness, breath work, self-compassion):

Oh no, I could definitely see it being helpful to a lot of them [AYA patients with cancer]. I was never told the ideas, so I don’t think that’s the reason why...I think that’s why I never did it. I didn’t have the option. No one told me about that, so to me, as a kid, my always escape was TV and sleep. So that was my go-to thing.

patient 09

Parents also described being open to mindfulness:

That would be the best thing you have to give these children is lessons in meditation because from what I’ve witnessed with my son, even the nurses at [hospital name], they were to a point just where everything they gave him wasn’t working.

parent 06

Health care providers also overwhelming endorsed the idea of a mindfulness-based program, stating that they believed it could equip AYA with new coping skills. One provider described how an app could be a valuable delivery method for mindfulness content:

Yes, I think that I’m personally a huge believer in meditation and mindfulness as a way of coping and I think that it would be really helpful. I have found that ways that are easy and accessible that are already being used, so say if a patient really likes an app or likes the format and goes on it anyway as a way to get support, to also have meditation in that can help to get that by.

provider 02

##### Main Theme 3: Desire to Connect With Other Cancer Patients

Almost all AYA participants described a desire to connect with other AYA with cancer. Many AYA described their belief that only other patients who had gone through the life-changing experience of cancer could understand them.

Well, there’s a level of camaraderie that you can’t get anywhere else. It’s like you know exactly what the other person went through without having to talk about it. It’s so much less energy and effort to spend time with a person like that.

patient 07

Most patients described how changes in their body had led them to feel differently about themselves, and many expressed enthusiasm for connecting with other AYAs to discuss these changes. For example, one young woman described interest in meeting other AYA through Facebook:

You know, [if I] met these girls, like, through the Facebook page, and I can ask them, like, oh, has your menstrual cycle came back? Like, oh, no, my menstrual cycle hasn’t come back. You know, stuff like that, because it’s like as a girl you ask yourself, like, is my menstrual cycle ever going to come back again?

patient 02

In fact, patients, parents, and providers were enthusiastic about the internet as a tool for facilitating peer-to-peer support. As one mother described:

Yes, and that has been one of the most incredibly beneficial things about it. If you had asked me before my daughter had cancer that my kid would be on the computer talking with people I don’t know at midnight, I would have flipped out. I would have never thought that would be the case, but I will tell you with her having continual setbacks with her situation drawing her away from her real friends in real life, this has been a way for her to communicate with people, and it has been amazing.

parent 05

One provider described her interest in social media as a tool for promoting social support:

There are a large percentage of these patients who will require multiple rounds of therapy and many of these patients will not be cured and they will succumb to their disease. I think that this population would benefit from a social networking or peer-to-peer type of outreach to help them sustain them through this journey.

provider 01

Main themes associated with AYA intervention preferences are presented in Table 2.

#### Integration of Framework and Key Interview Themes into Development of the Program

Examination of the RIM-based [24,25] interview themes guided the development of the Mindfulness for Resilience in Illness program content. Specifically, a persistent theme of anxiety related to uncertainty surrounding reoccurrence and treatment adverse effects confirmed the utilization of a mindfulness-based program. Qualitative data revealed that imaging scans are a source of anxiety, and one participant expressed concern about using the term “body scan” to describe a mindfulness activity. As such, we did not use this specific exercise and reviewed program content for other potential procedure-related terminology. In addition, discussion of self-image concerns informed our decision to end the program with an emphasis on self-compassion.

The strong interest of the AYA in connecting with and learning from other AYA who have experienced cancer guided the content development for both the mindfulness app and the Facebook-based social support group. Given that AYA were interested in—but mostly unfamiliar with—mindfulness, we decided to create videos featuring two sarcoma survivors as program hosts. Facebook group refinement included tailoring activities to promote group interaction and ensuring that group participation would be private.

Building on the interview results, the Learning to BREATHE program [27] informed the structure of the mindfulness app portion of the program. The Learning to BREATHE [27] program was chosen because it is a sequenced mindfulness program appropriate for introducing AYA to mindfulness. Learning to BREATHE has been shown to affect positive outcomes in high school [27,34] and college-aged students [35].

Provider interviews indicated that providers wanted to support their patients’ use of a mindfulness-based program. Providers also indicated that providers focused on addressing psychosocial needs of AYA (eg, psychologists, social workers, and child life specialists) would be most likely to implement the program. Collectively, these findings guided the development of a provider e-book guide.

All program content was reviewed by the study team to ensure that the content would be developmentally and cognitively appropriate for the intended participant sample. Intervention components are described in the aim 2 section; Textbox 1 and Table 3 present the app program content and a description of the Facebook group content, respectively.

**Table 2:** Suggestions from adolescents and young adults for the intervention.

Theme	Example quote
Positive tone	“I think that, and just like spreading positivity on that page is obviously going to be a huge plus”.
Privacy	“I don’t necessarily need this friend that I knew in elementary school to know that I am part of this sarcoma support group. Having that either be secret or specifically just within the app, I think that make some people feel more comfortable.”
Space to exchange practical tips for dealing with sarcoma	“Different sections dedicated to patients going through what they’re going through...What do you guys do when it’s scan time? How do you guys relax.”
Bright, appealing visuals	“I would say, go kind of colorful. Not over the top, but definitely a lot of colors are always fun to see. Appealing to the eye.”

**Table 3:** Description of Facebook content, average response rates by AYA participants, and sample posts and responses from among the 38 total Facebook posts.

Post type and sample post		n (%)	Response rate, n (%)	Sample reply
**Informational**		**7 (18)**	**N/A**	
	At the beginning of each week I’ll post a link to the weekly blog posted on the app. Check out this week’s blog post! [link to blog post URL]			
**App feedback**		**12 (32)**	**8 (47)**	
	Which relaxation from the program have you enjoyed the most, and why? (1) Focus your Attention, (2) Belly Breathing, (3) Finding an Anchor, (4) Fuller, Deeper Breath, (5) 1.2.3. Breathe (6) Sense the Body, (7) Happy Breath			“Belly breathing was my favorite because it really helped me calm down”; “I liked the finding an anchor because it focused on different ways to relax other than breathing (even though breathing relaxes me)”
	What did you think about today’s “Reflect and Redirect” exercise?			“It was very interesting, definitely going to try it when I need to next”; “I liked this one but I preferred the other two where we would name the emotion a bit more. The others where a bit more helpful to me since the reflection often caused me to start over analyzing and I wasn’t really able to get out of it.”
	It’s the final day of the program. List 4 words that describe how you feel about the program. What have you learned? Post a word, picture or image that helps to summarize what you have learned.			“I have learned to be calm and 4 words are calm passion thankful and nature”; “Introspection, breathing, calm, and accepting. I’ve learned that this is something that truly helps and that I should continue doing in some way, even if it’s only 5-10 minutes a day.”
**Interaction and reflection**		**19 (50)**	**9 (53)**	
	What is your spirit animal and why? (It doesn’t have to be an actual animal, you can channel your favorite singer/artist, etc) Give us a visual! Post a photo to illustrate!			“My spirit animal would be a lion. The lion is my spirit animal because it is stronger, courage(ous) and a leader. I’m very much like a lion…and they are also my favorite animal”; “Batman is my spirit animal. I believe Batman is someone that represents the good and bad in everyone, He transcends the limits of man. I believe that in battling cancer we all find ourselves becoming Batman!”
	You have $500 and you can shop for anything that affects your self-image. Post a picture of what you would buy.			“The $500 would go into my cosplay fund. I can buy fabric and wigs for characters I want to become”; “I think I would save it for my college fund. I feel like that would help my self-image in that it makes me more secure in my future and gives me more power and choice over it.”
	This is the last week of relaxation exercises, and the theme is positivity. For what in your life do you feel most grateful?			“I’m so grateful for my family, as cliche as that sounds”; “I’m most grateful for my life because after battling cancer and not knowing whether I would win or lose I’m grateful for every moment!”

### Aim 2: Pilot Test of the Mindfulness for Resilience in Illness Program

#### Characteristics of Participants

Of the 26 patients approached, 22 patients were screened. Of those not screened, two were lost to follow-up, one was not interested, and one was not available during the intervention period. Of the 22 AYAs screened, 22 were eligible and 20 enrolled in the study. One patient indicated interest and was invited to enroll but did not complete online consent forms. The other patient declined because of disinterest. Six providers were screened, found to be eligible, and enrolled. In total, 26 people participated in the pilot study. Twenty AYA participants consented to be in the study. Seventeen of these participants (mean age 19.1, SD 3.7 years; 41%, 7/17 female) downloaded the app and joined the Facebook group (seven of these participants participated in the aim 1 interviews). Of the remaining three participants, one formally withdrew, and two did not participate in the study after completing the online consent and prestudy questionnaires. Data presented throughout this report exclude these three individuals who withdrew from the study. Six additional health care providers consented to participate in the pilot study stage; all six were female. Based on information gathered in the earlier interviews about who would be most likely to offer a psychosocial intervention to patients, we purposefully recruited three child life specialists: two nurses and one psychologist. See Table 4 for details about participant characteristics.

**Table 4:** Characteristics of the adolescents and young adults (AYA) and providers participating in the pilot test (aim 2).

Characteristic		AYA (n=17)	Providers (n=6)	Total (N=23)
Age (years), mean (SD)		19.1 (3.7)		
**Age range (years), n (%)**				
	13-17	7 (41)		
	18-25	10 (59)		
**Sex, n (%)**				
	Male	10 (59)	0 (0)	10 (44)
	Female	7 (41)	6 (100)	13 (57)
**Ethnicity, n (%)**				
	Hispanic/Latino	8 (47)	0 (0)	8 (35)
	Non-Hispanic/Non-Latino	8 (47)	6 (100)	14 (61)
	Unknown	1 (6)	0 (0)	1 (4)
**Race, n (%)**				
	White	9 (53)	6 (100)	15 (65)
	Black/African American	1 (6)	0 (0)	1 (4)
	Asian	1 (6)	0 (0)	1 (4)
	Multiracial	6 (35)	0 (0)	6 (26)
	Unknown	0 (0)	0 (0)	0 (0)
**Treatment status, n (%)**				
	On treatment	1 (6)		
	1-3 months posttreatment	1 (6)		
	4-8 months posttreatment	4 (24)		
	1-2 years posttreatment	4 (24)		
	3-6 years posttreatment	6 (35)		
	Off treatment for unknown duration	1 (6)		
**Providers’ specialties, n (%)**				
	Oncologist		0 (0)	
	Nurse or medical assistant		2 (33)	
	Child life, psychology		4 (67)	

#### Mindfulness App Usage

On average, participants completed a mean 16.9 (SD 11.9, range 0-28) of the 28 unique sessions, used the app for a mean 10.2 (SD 8.2, range 1-23) days during the evaluation period, and engaged with the app for a mean 112.5 (SD 79.4, range 1-208) minutes.

#### Facebook Social Support Group Usage

All but one person (16/17) in the Facebook group posted at least one reply to the moderator’s prompts. The mean number of responses was 15.2 of 31 (49.0%, range 0%-96.8%); therefore, participants responded to approximately half the moderator’s posts. The daily response rate ranged from 23.5% to 75.0% (mean 49.9%); which means on any given day approximately half the participants responded to the moderator’s post. See Table 3 for a sample of the daily moderator prompts, example responses, and the mean response rates for each category of post.

#### Mindfulness App Acceptance

Overall, participants responded that they enjoyed using the app (mean 5.69, SD 1.12; 1=not at all to 7=very much so), would be likely to continue using the app (mean 5.13, SD 1.54; 1=definitely no to 7=definitely yes), and would be very likely to recommend it to others (mean 6.19, SD 1.05; 1=definitely no to 7=definitely yes). See Table 5 for the ratings of specific features.

**Table 5:** Results of acceptance tests completed by adolescents and young adults (AYA) and providers.

Question			Mean (SD)	Rated ≥5, n (%)
**AYA^a^**				
	How much did you enjoy using the prototype mindfulness mobile app?		5.69 (1.20)	13 (81)
	Overall, how easy or difficult did you find it to navigate through the mobile app?		5.50 (1.27)	12 (75)
	**How much did you like or not like the seven audio relaxations offered** **during...**			
		Week 1	5.19 (1.64)	12 (75)
		Week 2 (n=14)	5.00 (1.30)	9 (64)
		Week 3 (n=15)	5.07 (1.71)	8 (53)
		Week 4 (n=14)	5.14 (1.29)	8 (57)
	Recalling the weekly video introductions and conclusions, how much did you like or not like the videos of the program hosts?		4.56 (2.22)	9 (56)
	**How helpful were the following features** **...**			
		Facebook group	4.56 (1.75)	11 (69)
		Blog posts (n=13)	4.15 (1.46)	6 (46)
		Sleep meditations (n=13)	5.46 (1.76)	11 (85)
		Host videos (n=15)	3.80 (2.04)	8 (53)
	Did the mobile app help you to relax?		5.75 (1.39)	12 (75)
	Did the mobile app help you to learn about managing difficult thoughts and feelings? (n=15)		4.13 (1.64)	4 (27)
	Did the mobile app help you to learn about practicing self-kindness? (n=15)		4.93 (1.83)	7 (47)
	Did the Facebook group help you to connect with other people?		3.50 (1.97)	5 (31)
	If it was available to you, would you continue to use the mobile app?		5.13 (1.54)	10 (63)
	Would you recommend the mobile app to someone you know with cancer?		6.19 (1.05)	14 (88)
**Provider (n=6)**				
	How helpful do you believe the app may be for AYA with cancer?		6.67 (0.52)	6 (100)
	How likely would you be to recommend the mindfulness app to a family with an adolescent or young adult managing cancer?		6.83 (0.41)	6 (100)
	Overall, do you believe the sample Provider Guide would be a helpful resource for providers, or members of their teams, working with AYA with cancer?		6.50 (0.84)	6 (100)
	How useful do you think mindfulness-based stress reduction strategies are for AYA with cancer?		6.50 (0.55)	6 (100)
	How likely would you be to recommend the Mindfulness for Resilience in Illness program to another health provider?		6.67 (0.82)	6 (100)

^a^For AYA, n=16 unless otherwise noted.

**Participants’ recommendations for improving the mindfulness app.**

**Adolescents and young adults**

- Additional features: search bar, chat forum/support group, relaxing music, a place to write down thoughts, other activities (brain quizzes, etc)
- An online counselor or someone to talk to
- Making the videos a little shorter
- Improved navigation
- Change the audio narration to include fewer pauses
- Talking more about cancer throughout the app content, not just during the introductions and takeaway videos
- Decrease the number of passive activities (eg, audio/video) and increase the number of active activities (eg, journal)
- Integrate the social support group into the app; including more group conversations
- Meditations should keep running, even if the phone was in sleep mode; or add a sleep timer
- Enable commenting on specific exercises within the app

**Providers**

- Remove the dashboard feature for tracking progress
- More easily separate app into its different components (blog, video, mindfulness activities)
- More content featuring peers
- Add pre and post mood assessments into each exercise to help the youth notice benefits
- Continue to receive feedback from the patients
- Interactive chat rooms; someone available to respond in times of crisis; hotline link
- Add mood and sleep tracking

Open-ended responses about the experience of AYA with the mindfulness app revealed that some participants found the audio meditations to be relaxing, and in some cases detailed how they were helpful. As one male explained, “I loved that it helped remind me to be mindful of my actions that contribute to battling my anxiety” (patient 205). Others explained that the audios helped with sleep; one female said, “I sometimes have [a difficult time] time sleeping or getting a full night rest but after listening to the voices I slept through the whole night” (patient 206). Participants also offered a range of suggestions for improving the mindfulness app, which centered around improving navigation, more opportunities for active participation, and more references to the cancer experience. Recommendations are listed in Textbox 2.

#### Facebook Social Support Group Acceptance

Participants responded that they found the Facebook group somewhat helpful (mean 4.56, SD=1.75; 1=not at all helpful to 7=very helpful). All 16 participants who completed the acceptance test replied “yes” when asked, “Would you recommend that this type of online social group be part of a future support program for youth with cancer?”

Participants described their experience with the Facebook group in open-ended responses. Participants felt strongly that online social support was a crucial and absent component of their cancer experience. As one young female described, “A social group for young people affected by cancer is something major that is missing for the emotional side of treatment” (patient 215). Some participants stated appreciation for the group; for example, “I liked that it was a safe place to expose your own thoughts/anxieties” (patient 205). In addition, they appreciated that the questions posed by the moderator were not only direct questions about the app program; for example, “I liked that there were not just questions referring to the meditations” (patient 213). Some responded that being expected to respond daily was too much; for example, “That there were questions every day, it was a little hard to keep up with responding” (patient 209).

Participants also offered several useful suggestions for future iterations of the online social support group. To enrich the experience, a few participants suggested facilitating more meaningful relationship-building conversations. For example, a young female said:

I would recommend it, but also something slightly less faceless if that makes sense. It was difficult to actually get to know people since most of the questions or discussion topics were more “fun fact” than really getting to know. Maybe having some more personalized chat features or encouragement to post not just on prompts but on our own would also be good.

patient 213

**Recommendations from adolescents and young adults for improving the Facebook group.**

- Increase interaction among participants, including sharing diagnoses and situations, more personal conversations, talking about being in the hospital
- Opportunities to meet up with people
- Expand to other hospital patients
- Add inspirational quotes
- Pair up someone in remission with someone currently going through treatment
- Talking more about cancer and treatment instead of just emotions and exercises
- Asking people in the group to do things that take their mind off their issues (more talking to people, exercising, new hobbies, etc); education/advice about these types of things you can do to feel better
- Combining the questions with optional writing exercises
- Include some articles that don’t just pertain to emotional dealings but pragmatic tips for dealing with nausea or hair loss (like those found in magazines)
- Make the group easier to access within Facebook
- Extension of the blog posts, including having teens and young adults submit questions, topics, writing pieces, etc

Another female suggested that content could be more cancer-specific:

Articles that don’t just pertain to emotional dealings but pragmatic tips for dealing with nausea or hair loss. Something similar to how magazines give tips and tricks since that is a very palatable format to many people that I know, and something that I really missed from a lot of coping and support forums that I’ve encountered for cancer. It also makes the situation feel less momentous and alien since if it’s presented in such a way, ultimately leading to a certain amount of normalization that I know that I often seek out.

patient 213

Suggestions for future enhancements are summarized in Textbox 3.

#### Psychosocial Measures

Results of paired samples *t* tests indicated there were no significant differences from pretest to post test on measures of perceived social support, mindfulness, body image, or quality of life. Means are presented in Table 6.

#### Provider Acceptance of the Mindfulness App and Facebook Social Support Group

Providers were enthusiastic about the program. Asked if the mindfulness app would be very helpful for AYA with cancer, four of six responded 7, and two of six responded 6 (where 1=not at all helpful to 7=very helpful). Five of six responded 7, and one of six responded 6 when asked if they would be very likely to recommend it to a family with an adolescent or young adult managing cancer. Please see Table 5 for other quantitative responses.

Providers’ open-ended responses about the mindfulness app and the Facebook social support group also revealed enthusiasm for the approach. One provider reported that the peer-to-peer resources were important (eg, “Peer videos were great! I think you get the most buy in from teenagers when they can hear directly from others who have similar experiences”). Others described the importance of helping AYA to identify and regulate emotions, for example, “I also specifically liked the information about the temporary state of emotions” (provider 02) and “Love the ride the waves quote. This is so true. We cannot take away our emotions, but we can learn to deal with them” (provider 03).

#### Provider Acceptance of Provider Guide

Providers also reported that the provider guide would be a helpful resource (four of six chose 7, one of six chose 6, one of six chose 5; 1=not at all helpful to 7=very helpful), and that the program overall was something that they would be very likely to recommend to another health care provider (five of six chose 7, one of six chose 5; 1=not at all likely to 7=very likely).

**Table 6:** Psychosocial measures completed by adolescent and young adult participants.^a^

Measure	Preintervention, mean (SD)	Postintervention, mean (SD)
Social support	14.4 (3.9)	13.6 (3.4)
Mindfulness	24.9 (7.1)	23.7 (6.6)
Psychological functioning	6.1 (3.1)	6.6 (3.8)
Body image	3.9 (2.9)	3.0 (2.9)

^a^Data presented are for the 16 participants who completed pre- and poststudy questionnaires.

**Providers’ recommendations for improving the provider guide.**

- Additional resources; links with local resources
- Written and visual instructions for navigating the app (for providers who are less tech savvy)
- Make it available as an app for reference; have it available to parents as a resource

To aid with future efforts, providers were asked what information they would find most useful for introducing the mindfulness app to patients. Most (four of six) responded that they would like to receive the provider guide paired with video training on relaxation and mindfulness basic practice. Three providers responded that they would like to receive the provider guide paired with a live webinar run by an expert in mindfulness; two responded that they would like to receive the provider guide paired with a list of practitioners who could use it to help support patients. Suggestions for improvement are summarized in Textbox 4.

## Discussion

The aim of this study was to use a patient-centered approach to develop and pilot-test the Mindfulness for Resilience in Illness program, a mobile-based mindfulness and social support program for AYA with sarcoma and survivors. Overall, AYA used the program and responded positively to it, offering useful suggestions for improvement. This study extends the small body of research on the use of mindfulness-based programs for AYA with cancer [17], offers further support for the use of Facebook as a tool to offer social support to young people managing a health condition [36], and suggests that it may be acceptable to deliver this content through a mobile device to AYA who have experienced sarcoma.

Participants in our study were encouraged to complete one meditation per day for 28 days. On average, participants completed 17 unique meditations and used the app for 112 minutes over 10 of the 28 days, which suggests that AYA users may prefer informal use of mindfulness and resiliency skills rather than directed daily use. Limited data exist on how much time participants need to practice mindfulness meditations to experience meaningful change. Although many mindfulness apps exist, the quality and systematic examination of these apps vary. A recent review and evaluation of mindfulness-based iPhone apps [37] identified five apps that provided progressive/program-based mindfulness training with recommendations for daily practice. Of these, the Headspace app received the highest average quality score. In a recent study [38] of the effectiveness of the Headspace app, completion of 10 approximately 10-minute sessions, was found to be sufficient to positively impact stress, affect, and irritability. These 10 sessions were completed over 15.8 days. Although these app engagement data are comparable to the results from this study, the Headspace intervention was conducted with healthy adults so these findings may not generalize to a clinical AYA population. More research is needed to determine the appropriate mindfulness app treatment engagement dosage necessary to affect meaningful clinical change.

Acceptance data suggested that most participants enjoyed using the program and that it helped them to relax. Participants also suggested modifications that could improve the experience, such as including music in the app or including a timer so that the app could turn off after the user had likely fallen asleep. Practical suggestions such as these could help increase engagement in a program. Sleep difficulties are common among AYA with cancer [39], so it is important to get feedback on modifications to the app that might help with sleep. Given that others have reported that mindfulness may be helpful for AYA dealing with the experience of cancer [17], the main implication from our study is that it may be feasible to deliver an MBI to AYA with sarcoma using mobile technology. Mobile-based interventions can be used to complement or extend the benefits of in-person programs. As such, a program such as the one described in this paper may be a valuable complement to a hospital-based mindfulness program for AYA dealing with sarcoma.

Participants in the study were also encouraged to participate in a Facebook group with daily prompts to answer questions about the mindfulness app, or questions designed to promote positivity and social interaction. Participants responded to approximately half of the prompts, and approximately half of the participants joined the conversation on any given day. Quantitative acceptance data revealed that most participants did not agree that the Facebook group helped them connect with other patients. The qualitative feedback helped to explain this finding; AYA reported that they wanted more of the conversation to be about the cancer experience and they wanted more opportunities to get to know the other participants. These qualitative findings are consistent with results from surveys suggesting that AYA with cancer have unmet social needs [12] and suggest that improvements to a future Facebook-based social support group could be to facilitate more opportunities for relationship building among participants. Possibilities include crafting daily prompts specifically around the experience of living with cancer as a way to facilitate more meaningful conversation and deeper connections, and asking participants to participate in “buddy” exercises with another member of the group. In addition, guidelines for using Facebook groups in the management of disease include understanding the potential role of group “champions” [23]. In our experience, it was important to recruit a group moderator who was enthusiastic about the goals of the group and who had also experienced cancer. The daily presence of the moderator and the moderator’s willingness to respond to participants’ comments appeared to encourage conversation.

Finally, health care providers were asked to review a provider guide and comment on the program overall. The response was positive, and providers gave practical suggestions for improvement. Specifically, providers reported that including resources to help them learn mindfulness skills would be useful. For the pilot study, we purposefully recruited providers who would be likely to offer the program to AYA (based on findings from the preliminary interviews). As such, we are encouraged that this group of six providers saw value in the program.

We did not see a significant change in the psychosocial measures of mindfulness, social support, psychological functioning, and body image. The main goals of the study were to develop and evaluate the use and acceptance of a pilot program. Given that the examination of psychosocial outcomes was exploratory in nature, this sample may not have been adequately powered to detect significant changes in these outcomes. The pilot study also did not include an assessment of factors that may have affected psychosocial outcomes. For example, it was beyond the scope of this pilot study to assess whether treatment engagement factors (eg, adherence, type of sessions completed, mindfulness practice, Facebook engagement) affected outcomes. In addition, the intervention improvement recommendations may provide some insight into intervention-related factors that may have contributed to psychosocial outcome results. Specifically, the program content may not have included enough opportunities for social interaction and social discussions surrounding different aspects of cancer treatment. Collectively, given these findings and methodological considerations, more work is needed to determine the effects of this intervention on psychosocial outcomes.

There are several limitations to our study. Our sample included patients with sarcoma, and most patients were off treatment, which may limit the generalizability of our findings to AYA patients with other cancer diagnoses and at different phases of treatment. In addition, it was beyond the scope of this study to examine how gender or age may affect intervention outcomes. Although qualitative themes were consistent across participants and participants expressed a desire for advice from teens and young adults, future work would benefit from examining whether developmental stage and gender affect psychosocial treatment needs and outcomes. Feedback from this sample will inform future program refinement, and future studies will utilize randomized controlled trials to examine the effects of this intervention on psychosocial outcomes in more diverse samples of AYA with cancer.

This study used a patient-centered approach to evaluate the unique needs of AYA with sarcoma and develop a mobile-based mindfulness and social support program for this AYA patient population. Mobile-based programs are cost-efficient, easy to disseminate, and have wide reach. These findings provide preliminary evidence of the feasibility and acceptability of a mobile-based mindfulness and social support program for AYA with sarcoma.

## Abbreviations

AYA: adolescents and young adults
CAMM: Child and Adolescent Mindfulness Measure
IRB: Institutional Review Board
MBI: mindfulness-based interventions
RIM: Resilience in Illness Model
